# Complete genome sequence of *Treponema medium* isolated from foot of bovine digital dermatitis in Japan

**DOI:** 10.1128/mra.00656-25

**Published:** 2025-08-27

**Authors:** Akira Fukuda, Takashi Murakami, Noritsugu Abe, Yasuhiko Suzuki, Chie Nakajima, Masaru Usui

**Affiliations:** 1 Food Microbiology and Food Safety Unit, Division of Preventive Veterinary Medicine, School of Veterinary Medicine, Rakuno Gakuen University 13022, Ebetsu, Japan; 2 Farm Animal Surgery Unit, Division of Preventive Veterinary Medicine, School of Veterinary Medicine, Rakuno Gakuen University 13022, Ebetsu, Japan; 3 Total Herd Management Service, Notsuke, Hokkaido, Japan; 4 Division of Bioresources, Hokkaido University International Institute for Zoonosis Control 12810, Sapporo, Hokkaido, Japan; 5 International Collaboration Unit, International Institute for Zoonosis Control, Hokkaido University 12810, Sapporo, Hokkaido, Japan; 6 Division of Research Support, Hokkaido University Institute for Vaccine Research and Development 12810, Sapporo, Hokkaido, Japan; 7 Vaccinology for Clinical Development, Hokkaido University Institute for Vaccine Research and Development, Sapporo, Hokkaido, Japan; The University of Arizona, Tucson, Arizona, USA

**Keywords:** *Treponema*, bovine, digital dermatitis, complete genome

## Abstract

*Treponema* species are the primary causative bacteria of bovine digital dermatitis. This report describes the isolation and complete genome sequencing of *Treponema medium* strain T1 isolated from a hoof sole swab of a bovine in Japan. Genome sequences were obtained using a combination of long- and short-read sequencing technologies.

## ANNOUNCEMENT

Bovine digital dermatitis (BDD) is an infectious skin disease of the foot that leads to reduced dairy farm productivity. Molecular studies have identified *Treponema* species, particularly *T. medium*, *T. phagedenis*, *T. pedis*, and *T. denticola*, in BDD lesions (1, 2). Culturing and isolating *Treponema* under laboratory conditions is challenging because of their poor growth and the presence of other fast-growing bacteria in BDD lesions (3, 4). Studies on the isolation and complete genome analysis of bovine *Treponema* are very limited.

*T. medium* strain T1 was isolated from swabbing the plantar aspect of the interdigital cleft of a bovine exhibiting BDD symptoms (stage M2) in Hokkaido, Japan, in 2024 (5). The swab was transferred to the laboratory using an Anaerobic porter II (Terumo, Tokyo, Japan) on the day of sampling and cultured in pDDTp broth at 4°C for 3 days (3). The culture was then spotted on membrane filters (25 mm diameter and 0.45 µm pore) placed on fastidious anaerobe agar (Neogen, Lansing, MI, USA) supplemented with 5% defibrinated sheep blood, 10% fetal calf serum, and 5 mg/L each of rifampicin and enrofloxacin. After 6 h, the membrane was removed, and the agar was incubated at 37°C for 14 days (4, 6). Strain T1 was subsequently sub-cultured in mNOS medium at 37°C for 5 days, and bacterial species were identified using a phase-contrast microscope and PCR (7). The sub-culture was stored at –80°C in 15% glycerol stock (8, 9). For DNA extraction, the strain in glycerol stock was cultured using mNOS at 37°C for 5 days. All isolation procedures were performed under anaerobic conditions.

Genomic DNA was extracted for short-read sequencing using the DNeasy Blood and Tissue Kit (Qiagen, Hilden, Germany). A DNA library was prepared using the Nextera XT Kit (Illumina, San Diego, CA, USA) and sequenced on a MiSeq platform (Illumina). The resulting 290 Mbp (∼93× coverage) from 534,610 paired-end reads (300 bp) were trimmed using fastp v0.24.1 (10).

For long-read sequencing, genomic DNA was extracted using the Quick-DNA HMW MagBead Kit (Zymo Research, Irvine, CA, USA). Library preparation was performed using the Rapid Barcoding Sequencing Kit SQK-RBK114.24 (Oxford Nanopore Technologies, Oxford, UK). Sequencing was conducted using MinION with a FLO-MIN-114 R10.4.1 flow cell (Oxford Nanopore Technologies) and MinKNOW software with a 72-h run time and no alterations to voltage scripts. The obtained 214 Mbp (∼68× coverage) from 53,348 reads (*N*_50_; 7,996 bp) were demultiplexed and adaptor-trimmed using Porechop v0.2.4 (https://github.com/rrwick/Porechop/releases/tag/v0.2.4), followed by quality filtered using Filtlong v0.2.1 (minimum length, 1,000 bp) (https://github.com/rrwick/Filtlong/releases/tag/v0.2.1).

Hybrid assembly of both read types was performed using Unicycler v0.5.1 (11), resulting in a single circular chromosome of 3,100,078 bp with a 43.8% GC content. Circularization was called by Unicycler. The bacterial species was identified as *T. medium* through average nucleotide identity (ANI) analysis using DFAST_QC (≥95% ANI in the type strain of *T. medium* ATCC799293 (GCA_000413035.1) (12, 13). The chromosome sequence was annotated using DFAST v1.3.0 (13). All software tools were used with the default parameters unless otherwise indicated.

## Data Availability

The whole-genome sequence has been deposited in DDBJ/ENA/GenBank under the accession number AP041040. The Illumina and MinION sequence reads were deposited in the Sequence Read Archive database under the accession numbers DRR684816 and DRR684817, respectively.
